# Personalized text message and checklist support for initiation of antihypertensive medication: the cluster randomized, controlled check and support trial

**DOI:** 10.1080/02813432.2020.1753380

**Published:** 2020-05-02

**Authors:** Aapo Tahkola, Päivi Korhonen, Hannu Kautiainen, Teemu Niiranen, Pekka Mäntyselkä

**Affiliations:** a Institute of Public Health and Clinical Nutrition, University of Eastern Finland, Kuopio, Finland;; b Health Centre of Jyväskylä Cooperation Area, Jyväskylä, Finland;; c University of Turku, Turku, Finland;; d Medcare Oy, Espoo, Finland;; e National Institute for Health and Welfare, Helsinki, Finland;; f Primary Health Care Unit, Kuopio University Hospital, Kuopio, Finland

**Keywords:** Checklist, drug prescription, hypertension, medication, newly diagnosed, target, text message

## Abstract

**Objective:** To assess whether the use of a checklist combined with text message support improves systolic blood pressure (SBP) control.

**Design and setting:** A cluster randomized controlled trial in Finnish primary care.

**Interventions:** Personalized text message support and a checklist for initiation of antihypertensive medication.

**Patients:** 111 newly diagnosed hypertensive patients aged 30–75 years.

**Main outcome measures:** The proportion of patients achieving 1) the office SBP target <140 mmHg or 2) the home SBP target <135 mmHg at 12 months.

**Results:** 28% (*n* = 16) and 31% (*n* = 17) of patients in the intervention and control groups met the office SBP target, respectively (*p* = 0.51). The corresponding proportions were 36% (*n* = 18) and 42% (*n* = 21) for the home SBP target (*p* = 0.21). Office SBP decreased 23 mmHg (95% CI: 29–17) in the intervention group and 21 mmHg (95% CI: 27–15) in the control group (*p* = 0.61). Medication changes, number of antihypertensives at 12 months and health care utilization were similar in both study groups. Patients considered checklist and text message support useful and important.

**Conclusion:** Only a small proportion of patients in the intervention and control groups reached their treatment target despite multiple health care contacts and medication changes. The study interventions did not improve SBP control. However, this study demonstrates new information about hypertension control, antihypertensive medication and health service utilization during the first treatment year.

## Introduction

Improving blood pressure (BP) control remains a major challenge for health care as most patients on antihypertensive medication fail to achieve their BP target [1,2]. Inadequate BP control leads to numerous preventable deaths and disabilities [3]. We need urgently novel ways of addressing barriers to successful hypertension treatment. This is especially true in primary care (PC) where the majority of hypertensive patients are treated [4].

The utilization of Short Messaging Service (SMS) is one relatively new approach to help more patients reach their BP target. SMSs are widely spread, low cost and easy-to-use even for older patients. Previously, SMSs have been demonstrated to enhance BP control successfully in some, but not all studies [5]. Therefore, we need better understanding about the optimal application of SMS support for improving BP control in real life PC setting.

A checklist for the initiation of antihypertensive medication (hereafter, checklist) might serve as a feasible way to personalize SMSs and probably further increase their impact. We have previously reported that checklist implementation improves hypertension treatment in short term [6]. Checklists may also enhance treatment compliance, at least in inpatient care [7]. However, it is unclear if using a checklist can enhance BP control in long-term follow-up and in PC setting. It is also unknown if the combined use of a checklist and SMSs would be more effective than either one alone.

The aim of this study was to investigate whether a personalized text message support, together with a checklist, would improve systolic blood pressure (SBP) control as compared with usual care during the initial 12 months of antihypertensive therapy.

## Material and methods

### Study design

The Check and Support Study (ClinicalTrials.gov reference NCT02377960) was a cluster-randomized controlled trial in Finnish PC setting. The study was conducted in accordance with the principles of the Declaration of Helsinki and the ethical standards of the institutional review board of the Hospital District of Northern Savo (reference 63/2014). Written informed consent was obtained from all the study patients. Study reporting is in line with Consolidated Standards of Reporting Trial (CONSORT) 2010 guidelines.

Eight PC study centers in Central Finland were recruited to take part in the study between 27 January 2015 and 6 March 2018. The study centers included five public sector health centers, one private occupational care center and one public sector health center that also provided occupational health care. All study centers in both arms received basic information about the study and a short lesson on current hypertension treatment guidelines. All the study centers were first grouped into comparable pairs and then randomized to function either as an intervention (*n* = 4) or control (*n* = 4) center. The pairing was done to match the following attributes: Center size (small–large), location (urban–rural), and selection of services (occupational health care service or not).

### Study population

Study patients were recruited by treating physicians during routine medical appointments when initiating a new antihypertensive medication. Inclusion criteria were: (1) age of 30–75 years, (2) initial antihypertensive prescription (3) a clinical diagnosis of hypertension, (4) having a mobile phone, (4) ability to read SMSs, (5) ability to take care of the personal medication, (6) ability to perform home BP measurements adequately and (7) an agreement to use electric drug prescriptions (standard care in Finland). Exclusion criteria were: (1) unwillingness to give informed consent and take part in the study, (2) a malignant disease that was determined to have an impact on life expectancy, (3) pregnancy, (4) atrial flutter or atrial fibrillation, (5) having or suspected of having depression or psychosis, (6) rapid onset or worsening of hypertension, (7) SBP > 200 mmHg, (8) DBP > 120 mmHg, (9) hypokalemia (*K* < 3.3 mmol/l) or (10) kidney disease, defined as an estimated glomerular filtration rate < 45 ml/min/1.73 m^2^, or proteinuria (albumin–creatinine ratio > 30 mg/mmol, night urine albumin > 200 μg/min, 24-h protein excretion > 500 mg/day, or urine dipstick test showing proteinuria).

### Interventions

**Figure A1.**
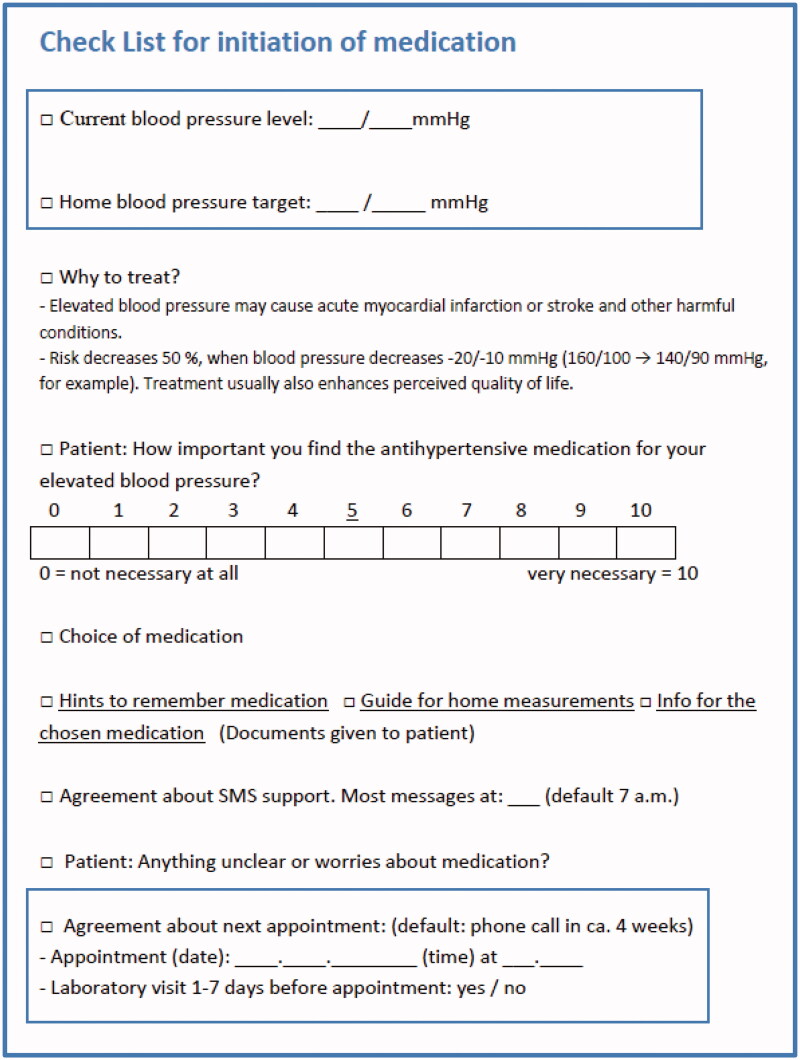
Checklist for initiation of medication. After filling in the checklist with the clinician, the patients received a copy of it for themselves, together with enclosed written information. Underlined sections refer to written information enclosed with the checklist. This information included five alternative medication guides depending on the physician’s choice. The figure was originally published in: Tahkola et al. [6] and distributed under the terms of the Creative Commons Attribution 4.0 International License (http://creativecommons.org/licenses/by/4.0/). No changes for the original figure were made.

The Information-Motivation-Behavioral skills (IMB) model was used as a theoretical model to design study intervention tools [8]. Initiation of medication in the intervention group (I-group) was carried out using a 9-item checklist (Appendix, Figure A1) filled in together by the patient and the treating physician [6]. The checklist aimed to help the treating physician to provide the essential motivational, informational and behavioural elements for successful hypertension treatment. For example, it reminded the physician to define an exact BP target and a personal treatment plan before the appointment was over.

**Figure 1.**
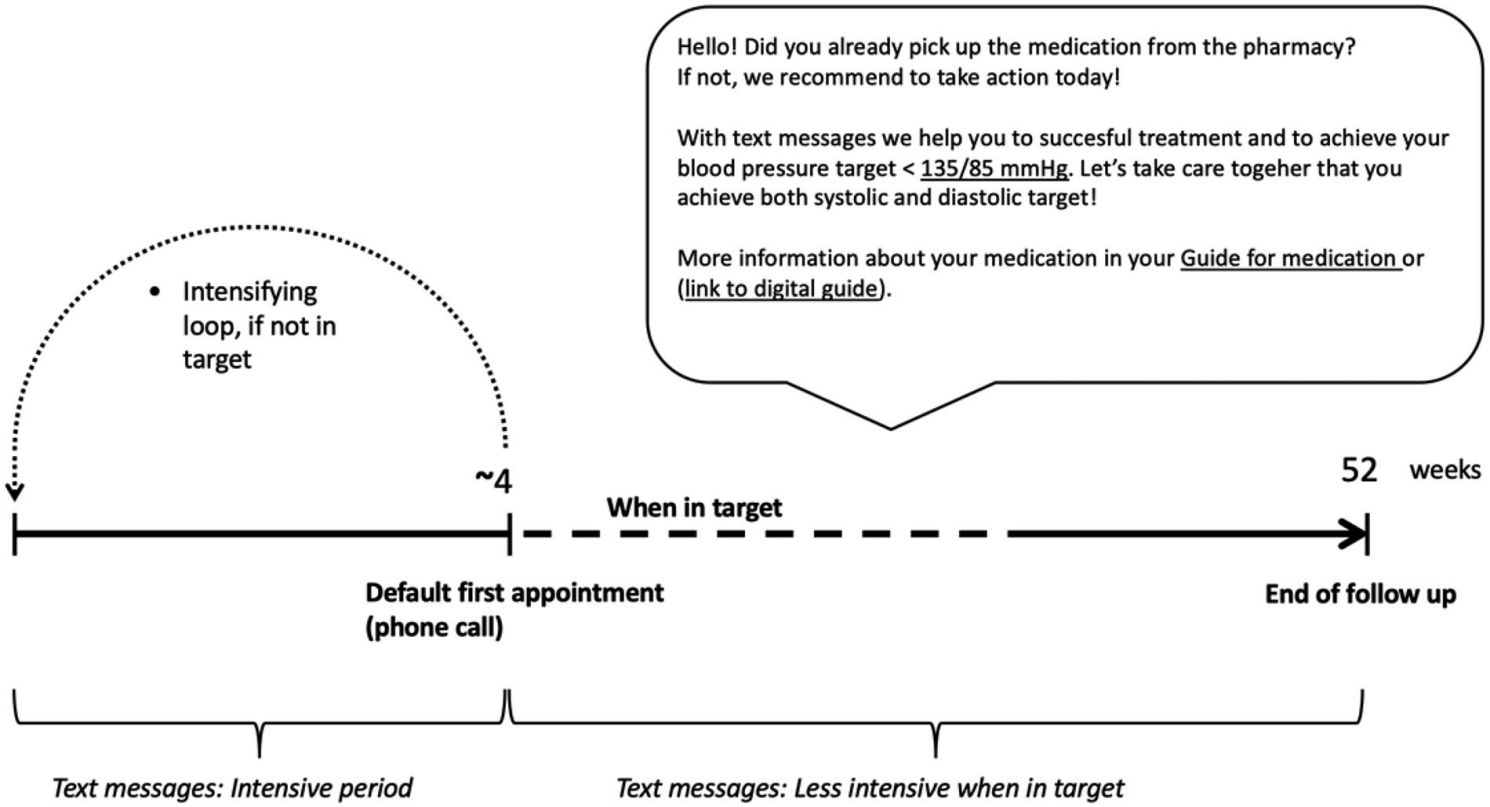
The timeline of text message support and a selected example of a text message. Underlined sections refer to personalised content received from the checklist of initiation of medication. Personalising was made in terms of timing, personal BP target and individual medication.

Information from the checklist was then used to personalise SMS support in terms of timing, personal BP target and individual medication. Default time for morning messages was 7 a.m. After appointment, a personalised, unidirectional SMS support was initiated for 12 months. Figure 1 shows the time line of SMS support and an example of a SMS. For the first 2 weeks, SMSs were sent on daily basis, focused on medication-reminders and coping with the potential side effects of medication. From third week on, SMSs were sent less often and they focused more on keeping up with the medication and remembering the importance of leading a healthy lifestyle, performing adequate home BP self-monitoring, achieving the personal BP target and attending clinical appointments.

The default for the first follow-up appointment was a phone appointment at about 4 weeks. However, the treating physician was allowed to modify the schedule. If the BP target was not reached at the first follow-up appointment and the treating physician decided to intensify antihypertensive medication, SMSs started again from the beginning. Thus, the patient received a new intense period of SMSs, tailored for the new medication and treatment plan. This loop of intensification could be repeated as many times as needed to achieve the personal target BP. However, the total duration of SMS support remained always 12 months. If more than two intensifying loops were needed, intensification was less intense to avoid information fatigue. Altogether, all patients received >43 SMSs.

Hypertension treatment in the control group (C-group) was managed by the treating physician without a study-specific protocol for treatment. No study-specific medication protocol was used in neither study arms.

### Measures and data collection

#### Measures and baseline characteristics

Office BP, waist circumference and weight were measured by treating physician. Office BP was measured three times from the left arm after five minutes of rest in the sitting position with a Microlife WatchBP Home A or N automatic oscillometric monitor [9]. Default cuff was a wide-range (arm circumference 22–42 cm) semi-rigid conical cuff. Large (arm circumference > 42 cm) and small cuffs (arm circumference < 22 cm) were also available. The same BP monitor was then loaned to the study patients to be used for home BP measurements. Both written and oral instruction for home BP measurements were given to all study patients.

Questionnaires on basic demographics, smoking habits (Heaviness of Smoking Index) and alcohol use with alcohol consumption questions (AUDIT-C from AUDIT) were completed [10,11]. Frequency-Intensity-Time (FIT) Index was used to assess exercise habits [12]. The score range of FIT index is 1–100; points <36 indicate low, 37–63 moderate and 64 or more high physical activity. A questionnaire also included questions on the three elements of the IMB model: informational, motivational and behavioral skills [6,8]. As part of the questionnaire at 12 months, patients in the I-group were also asked to evaluate the usefulness of SMS support (‘How useful did you find SMSs for supporting your treatment?’) and importance of the checklist (‘In addition to SMSs, you filled up a checklist with your treating physician to support your treatment. How important did you find it?’) with an 11-point numerical rating scale (0 = not useful or important at all, 10 = very useful or important). The willingness to receive hypertension treatment related SMSs in the future (‘Would you like to receive SMS support for your treatment in the future?’; Yes or No) was also evaluated.

An electrocardiogram (ECG) was taken and the following laboratory tests were performed: plasma potassium, plasma creatinine, fasting plasma glucose and fasting plasma cholesterol and estimated glomerulus filtration rate (eGFR) [13]. Proteinuria was measured with the albumin excretion rate measured from spot urine albumin–creatinine ratio, nightly urine, or diurnal urinary protein excretion.

Office BP was defined as the mean of three measurements. Home BP was defined as the mean of all measures over a 7-day period (three measurements twice daily, at 6–9 a.m. and 6–9 p.m.). College- or university-level education was considered higher education. Body Mass Index (BMI) was calculated by dividing the patient’s weight (kg) by the square of his/her height (m). Table 1 shows the baseline characteristics of the study patients.

**Table 1.** Baseline characteristics of the study patients according to study groups.

Characteristics	Intervention *n* = 59	Control *n* = 59	*p* Value
Female, n (%)	39 (66)	35 (59)	0.45
Age, years, mean (SD)	58 (11)	58 (10)	0.89
Higher education, n (%)	21 (36)	9 (15)	0.011
Married or co-habiting, n (%)	45 (76)	47 (80)	0.66
Working, n (%)	31 (53)	26 (44)	0.41
Diabetes mellitus, n (%)	5 (9)	7 (12)	0.54
Physical activity, FIT index, mean (SD)	40 (19)	36 (20)	0.35
Alcohol use, AUDIT-C index, mean (SD)	3.3 (2.7)	3.3 (2.5)	0.97
Smoking, Heaviness of smoking index, mean (SD)	9 (15)	11 (19)	0.62
Office SBP, mmHg, mean (SD)	172 (20)	173 (20)	0.87
Office DBP, mmHg, mean (SD)	101 (12)	102 (13)	0.72
Home SBP, mmHg, mean (SD)	156 (15)	152 (13)	0.20
Home DBP, mmHg, mean (SD)	91 (7)	93 (8)	0.50
Total cholesterol, mmol/l, mean (SD)	5.44 (1.17)	5.46 (1.13)	0.92
LDL cholesterol, mmol/l, mean (SD)	3.19 (1.02)	3.27 (1.10)	0.71
HDL cholesterol, mmol/l, mean (SD)	1.60 (0.47)	1.58 (0.47)	0.83
Triglycerides, mmol/l, mean (SD)	1.37 (1.42)	1.49 (0.70)	0.59
eGFR, ml/min/1.73m^2^, mean (SD)	89 (16)	91 (14)	0.52
Fasting glucose, mmol/l, mean (SD)	5.85 (0.91)	6.13 (1.25)	0.19
BMI, kg/m^2^, mean (SD)	28.9 (4.4)	30.5 (6.0)	0.10

University- or college-level education was considered higher education. AUDIT-C: alcohol consumption questions from the alcohol use disorders identification test (AUDIT); BMI: body mass index; DBP: diastolic blood pressure; eGFR: estimated glomerulus filtration rate (CKD-EPI equation); EQ-5D: EuroQoL questionnaire of health-related quality of life; FIT index: Frequency-Intensity-Time (FIT) Index; HDL: high-density lipoprotein; LDL: low-density lipoprotein; SBP: systolic blood pressure.

#### Outcomes and data collection

*Primary outcomes.* The office and home SBP targets were <140 mmHg and <135 mmHg, respectively, in accordance with the then-current European and Finnish Society of Hypertension guidelines [14,15].

*Data collection and secondary outcomes.* Study outcomes were collected during, or immediately after the final follow-up appointment at 12 months. All baseline measures were repeated. The study questionnaires were sent by mail to study patients prior to appointment, where they were collected and saved for analyses together with home BP measurements and home BP measurement device. Hypertension-related use of health care services and medication information, used as secondary outcomes, were assessed by examining participants’ electronic health records together with a questionnaire.

### Sample size

A power analyses based on the study hypothesis was carried out to determine a sufficient amount of patients. The study hypothesis was that the proportion of patients achieving the SBP target at 12 months would be 24% in the C-group based on studies of Finnish PC patients, and that the study interventions would improve the proportion to 50% [16,17]. The sample size was estimated using iterative models according to cluster randomization principles. We thus planned to recruit 140 study patients (70 in each group) in order to detect a significant difference with a power of 80% by the two-side α = 0.05. However, only 119 patients were finally recruited, due to a slower recruitment rate than expected.

### Statistical analyses

Data is presented as means with standard deviation (SD) and as counts with percentages. Statistical comparisons between groups were made using *t*-test for continuous variables and Pearson’s chi‐square for categorical variables. A bootstrap method was used when the theoretical distribution of the test statistics was unknown or in case of violation of assumptions (e.g. non-normality). To test the effect of intervention on blood pressure, we used a generalized linear mixed models with appropriate distribution and link function, and assuming a covariance structure as unstructured. Random effects accounted for within-practice clustering and within-patient correlation. Stata 16.0 (StataCorp LP; College Station, Texas, USA) statistical package was used for the analysis.

## Results

**Figure 2.**
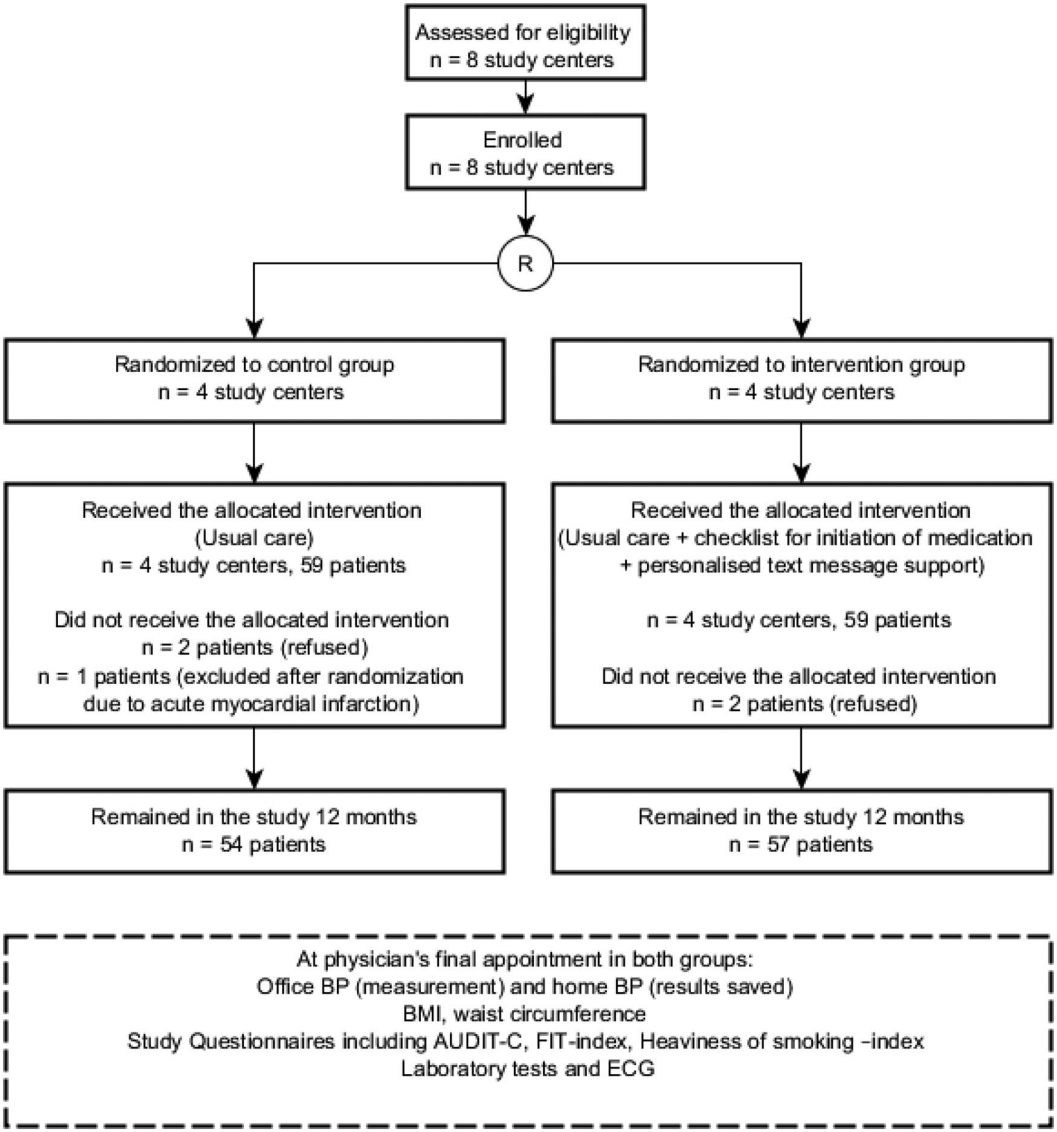
The flow of the study. AUDIT-C: alcohol use disorders identification test; BMI: Body Mass Index; BP: Blood pressure; ECG: electrocardiogram; FIT index: Frequency-Intensity-Time (FIT) Index; Laboratory tests: fasting plasma glucose level, fasting plasma cholesterol level, existence of proteinuria, creatinine level.

In total, 118 patients were included in the analysis and received allocated interventions. At 12 months, 111 patients had remained in the study (*n* = 57 in the I-group, *n* = 54 in the C-group) and were included in the analysis. Seven patients (6%) dropped out of the study. The study flow is presented in Figure 2.

During the follow-up, office SBP decreased 23 mmHg (95% CI: 29–17) in the I-group and 21 mmHg (95% CI: 27–15) in the Control-group (*p* = 0.61). Office DBP decreased 13 mmHg (95% CI: 16–9) in the I-group and 13 mmHg (95% CI: 16–9) in the C-group (*p* = 0.92). Home SBP decreased 18 mmHg (95% CI: 22–14) in the I-group and 13 mmHg (95% CI: 17–8) in the Control-group (*p* = 0.078). Home DBP decreased 10 mmHg (95% CI: 12–8) in the I-group and 10 mmHg (95% CI: 12–7) in the Control-group (*p* = 0.72).

**Figure 3.**
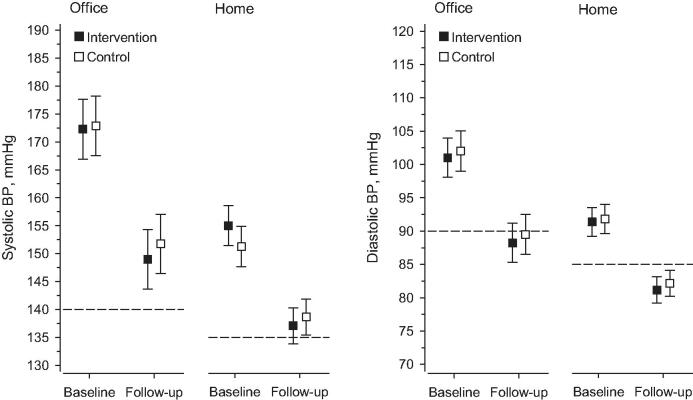
Blood pressure (BP) control and BP changes in two study groups during the 12-month follow-up. Dotted lines indicate BP target levels. The office systolic BP target was <140 mmHg and home SBP target was <135 mmHg. The office diastolic BP target was < 80 mmHg for diabetics and < 90 mmHg for others. For home diastolic BP, these targets were < 85 mmHg and < 75 mmHg.

At 12 months, 28% (95% CI: 17–42) (*n* = 16) of patients in the I-group and 31% (95% CI: 20–46) (*n* = 17) of patients in the Control-group reached the office SBP treatment target (<140 mmHg), with no between-group differences (*p* = 0.51). Adjustment for baseline age, sex and systolic blood pressure did not change the result. Home SBP target was reached by 36% (95% CI: 23–51) (*n* = 18) of patients in the I-group and 42% (95% CI: 28–57) (*n* = 21) of patients in the Control-group (*p* = 0.21). At 12 months, 30% of all study patients reached the systolic office BP target and 36% the systolic home BP target (Figure 3).

The most common primary antihypertensive medication was angiotensin II receptor blocker in the I-group (I-group) (*n* = 33, 56%) and in the Control-group (*n* = 30, 51%). Combination therapy was initiated for 13% (*n* = 14) of all study patients, of whom 10 patients were in the I-group. Altogether, 70% of all study patients underwent at least one medication change during the follow-up. The mean number of antihypertensive medication changes was 2.0 (range: 0–9) in the I-group and 1.4 (range: 0–12) in the Control-group (*p* = 0.088). Any medication change due to side effect of medication was made for 15 patients (26%) in the I-group and for 10 patients (19%) in Control-group (*p* = 0.33), of which only two were made for a patient with ARB monotherapy. At 12 months, the mean number of antihypertensives was 1.7 in the I-group and 1.4 in the Control-group (*p* = 0.17).

The mean number of healthcare contacts per patient was 5.5 (SD 3.3) in the I-group and 4.8 (SD 2.8) in the Control-group (*p* = 0.22). The most common method of contact was a phone call for treating physician in the I-group and office visit for treating physician in the Control-group. Patients in the I-group had more phone contacts with the treating physician (2.8 in the I-group, 1.5 in the Control-group, *p* < 0.001), whereas patients in the Control-group had slightly more nurse visits (0.1 in the I-group, 0.3 in the C-group, *p* = 0.037).

At 12 months, patients in the I-group considered SMS support useful. In an 11-point numerical rating scale (0 = not useful at all, 10 = very useful), the mean rating was 8.3 (SD 1.8). The checklist was also considered important with the mean rating of 8.5 (SD 1.6) in an 11-point numerical rating scale (0 = not important at all, 10 = very important). Of the patients in the I-group, 49% were willing to continue SMS support.

## Discussion

### Statement of principal findings

In this randomized, controlled trial, personalized SMS support combined with a checklist did not improve SBP control at 12 months in a PC setting. Despite multiple contacts with health care and numerous medication changes, only a third of all study patients reached their treatment target. However, positive feed-back about the study interventions and their neutral effect on health service utilization encourages us to investigate the applications of interventions further in PC settings. The study also demonstrates new information about hypertension control, antihypertensive medication and health service utilization during the first treatment year.

### Strengths and weaknesses of the study

Our study had some specific strengths. First, we carried out a randomized trial in a real life PC setting with a clinically relevant follow-up time of 12 months and low drop-out percentage (6%). To our knowledge, this is the first randomized controlled study to investigate SMS support during the first year of antihypertensive medication and the first trial combining SMS support with a checklist. Second, study patients are representative of typical hypertensive patients in PC settings and the study findings are therefore widely applicable to PC. Third, we recruited only newly diagnosed hypertensive patients while previous studies have concentrated on individuals with existing treatment for hypertension. This makes it easier for future investigators to consider study findings when designing new interventions alike. Fourth, according to our observations, the cluster-randomisation design protected our study effectively against the possible cross-over effect. Control centers did not adopt study interventions during the study period. Furthermore, our study had a strong theoretical basis (IMB model), which helps future researchers to further develop study interventions [8].

The study also had some limitations. First, we could not recruit as many patients as our pre-study power analyses suggested. This limited the statistical power of the study, but it hardly explains the lack of impact of the interventions, which may rather be due to clinical and therapeutic inertia. Despite short lessons on current hypertension treatment guidelines in every study center before onset of recruitment and the awareness of participating in a clinical study, treating physicians were often satisfied with inadequate BP control. Second, the study centers in C-group seemed to have focused on hypertension treatment more than usually with an average of five contacts to health care during the study period. Hence, the treatment also in C-group was probably more effective than usual. We believe, that the non-difference between the study groups is at least partly explained by this factor.

### Findings in relation to other studies

To our knowledge, there is no existing evidence on the impact of checklists on BP target reaching in PC. We demonstrated previously the immediate positive impact of checklist implementation on PC hypertension treatment [6]. Checklists have also been reported to enhance treatment compliance in inpatient care setting [7]. However, these findings did not translate into improved long-term BP control in our study.

Several studies have investigated the impact of SMSs on hypertension treatment. Recently, the StAR trial reported a small reduction (mean adjusted change of −2.2 mm Hg) in SBP at 12 months compared with usual care [18]. Kiselev et al. [19] reported significant increase in BP target reaching (77% in the I-group) due to utilization of SMSs, but high proportion of their study patients in the I-group (36%) were withdrawn from the study during the follow-up. Golshahi et al. [20] and Wald et al. [21] did not find significant change in BP levels in their studies.

### Meaning of the study: Implications for research and practice

Our results indicate a need for further research on the optimal applications of checklists and SMS support. Future studies should also investigate the most effective treatment strategies for the first year of antihypertensive medication. Our study results also strongly demonstrate how challenging the treatment of hypertension often is in PC settings, supporting previous research findings [22]. Even a relatively rigorous treatment scheme that includes several contacts with health care during the first treatment year does not guarantee good BP control in real life primary care setting. One reason behind this failure may be the low utilisation of combinations as primary antihypertensive medication despite relatively high baseline BP levels of study patients. However, we saw quite remarkable reduction in BP levels in both study groups indicating that current treatment strategies nevertheless translate into significant risk reductions. Our results also suggest that choosing an ARB (when suitable) for primary antihypertensive medication may help clinicians to avoid unnecessary medication side effects.
